# Quantitative Analysis of the Shape Characteristics for Quartz Particles

**DOI:** 10.3390/ma19061068

**Published:** 2026-03-11

**Authors:** Wenqing Jiang, Zhenggang Jia, Yanchang Jiang, Wenjie Yuan

**Affiliations:** 1 State Key Laboratory of Advanced Refractories, Wuhan University of Science and Technology, Wuhan 430081, China; jiangwenqing0528@163.com; 2 Hubei Annaijie Lining Materials Co., Ltd., Xiangyang 441100, China; jiazg16@sina.com (Z.J.); 13487131419@163.com (Y.J.); 3 National-Provincial Joint Engineering Research Center of High Temperature Materials and Lining Technology, Wuhan University of Science and Technology, Wuhan 430081, China; 4 Joint International Research Laboratory of Refractories and Metallurgy, Wuhan University of Science and Technology, Wuhan 430081, China

**Keywords:** quartz, particle shape characteristics, quantitative analysis, fractal dimension

## Abstract

Quartz, as an important non-metallic mineral, is widely used in many industrial fields. The shape characteristics of quartz particles are key factors affecting the bulk density, construction performance, and high-temperature performance of dry ramming mixes. This study focuses on quartz with seven different particle sizes from four different origins. Using digital image processing technology, geometric parameters such as flatness, circularity, and angularity of the particles (with a count equal to or exceeding 300 for each particle size category) were quantitatively analyzed, and the fractal dimension of quartz particles was calculated based on fractal theory. The results show that quartz from Luoyang exhibits the highest flatness, and quartz particles from Fengyang present the highest circularity, while the flatness and angularity of quartz particles from Xiangyang and Chengde are similar. The fractal dimensions of 20–100 mesh quartz particles from Fengyang are in the range of 1.027 to 1.060, greater than those of the other quartz particles. At smaller particle sizes, the shape of quartz from various origins tends to be regular (the circularity of particles of 70–100 mesh is >0.8). Through the quantitative characterization of parameters, the relationship between particle shape and size was revealed, which provided an important basis for selecting the raw materials and performing quality control for silica-based dry ramming mixes.

## 1. Introduction

Medium-frequency induction furnaces are widely used in foundries and nonferrous metal smelting due to their strong heating capacity, intermittent operation, flexible and convenient use, and other advantages [1,2]. Large-scale and high-power versions of these furnaces are being developed with the growth in their demand.

A cheap and rapidly replaceable acid lining is crucial for maintaining the production efficiency, energy efficiency, and service life of medium-frequency induction furnaces. Quartz, as the main raw material, is a mineral resource with excellent performance that is widely present in nature. The CO_2_ emissions from quartz are lower than those of calcined or fused raw materials and comply with environmental protection requirements. However, the purity and grain size of quartz depend on its origins [3,4], creating difficulties to raw material screening and lining quality control. Silica-based dry ramming mixes comprise quartz aggregates, fine particles, and powders; their quality depends on the grading, size, and shape of raw materials. Particle shape characteristics are also an important factor affecting lining service life under the same process. Therefore, the quantitative analysis of quartz is essential to ensure highly efficient utilization of natural minerals.

In general, the particle shape is often quantitatively described by parameters such as circularity, flatness, angularity, and fractal dimension [5,6,7,8] to make up for the lack of qualitative terms such as “spherical, acicular, and flaky” [9]. Quartz particles are polyhedral, and their shapes are irregular and random. They appear as irregular polygons in a two-dimensional projection. Their geometric properties (such as area, perimeter, Feret diameter, and equivalent circle diameter) have a direct influence on particle properties and stacking behavior. With the development of image analysis technology, the quantitative method of particle shape analysis has gradually evolved from manual measurements to digital characterizations using a scanning electron microscope, and three-dimensional scans, providing technical support for accurately describing particle shape [10,11].

In fractal geometry, the fractal dimension as a characteristic parameter can describe irregular and self-similar graphics [12,13]. Irregular particles are projected onto the plane to form an irregular plane geometry and the projection image of particles is analyzed based on fractal theory. In principle, the method includes variable and fixed sizes. The latter involves the methods such as perimeter–area [14], perimeter–maximum diameter, box counting, etc. The fractal dimension can be calculated by the classical perimeter–area method in fractal geometry theory. The particle shape distribution characteristics can be evaluated using the fractal dimension.

The correlations between particle morphology and the gradation of the shear strength of granular assemblies have been investigated in geotechnical and mining engineering [15], and the effect of particle shape on the undrained shear behavior of mixtures of sand and fine particles has been examined [16]. The factors with the greatest influence on the packing density of granular materials include the particle size distribution, particle shape and compactness, mixture quality, and container size [17]. It is possible to use shape parameters to estimate the packing and properties of dry ramming mixes, as is for granular assemblies.

The characteristics of quartzite vary significantly depending on its origin and batch. Despite the abundance of quartz resources, the selection and processing of raw materials for furnace linings must be guided by their specific properties. The aim of this study is to conduct a quantitative analysis of the shape characteristics of quartz particles with different sizes from various origins. Through the digital images of quartz particles obtained by an optical microscope, all aspects of shape characteristics, including flatness, circularity, angularity, and fractal dimensions, were evaluated to provide a basis for selecting the raw materials and performing the quality control of silica-based dry ramming mixes for the induction furnace lining.

## 2. Materials and Methods

Quartz from four different origins (Xiangyang, Hubei; Fengyang, Anhui; Chengde, Hebei; Luoyang, Henan) was selected and numbered A–D. Their chemical compositions are listed in Table 1. Coarse crushing of quartz was first performed using a jaw crusher (60A, Shanghai Shuli Instrumentation Co., Ltd., Shanghai, China) to break down large pieces of rocks into particles with a size less than 15 mm, which was then directed into the double-roller sand making machine (SG300*300, Shandong Ruinachuang Machinery and Equipment Co., Ltd., Tai’an, China) for further grinding. Finer particles (−20 mesh) were obtained through ball milling. Quartz particles of 4–6 mesh, 6–8 mesh, 8–10 mesh, 10–20 mesh, 20–50 mesh, 50–70 mesh, and 70–100 mesh were obtained by screening from four different origins. Microscopic photos of quartz particles were obtained via an optical microscope (Motic, SMZ-171, Xiamen, China). The digital images were processed using the software ImageJ 1.54g (Wayne Rasband, National Institutes of Health, Bethesda, MD, USA) to determine the contour perimeter, Feret diameter and particle projection area of the particles (two-dimensional). The resolution of the images was 3072 × 2048 pixels. Manual thresholding was carried out after image calibration using a standard scale bar, and characteristic parameters such as the flatness, circularity, and angularity were calculated. The statistical count of particles for each particle size category was equal to or greater than 300.

During the procedure, quartz particles were positioned on the substrate and imaged using suitable magnification. Image preprocessing is required to enable the effective statistical analysis of particle morphology. Initially, the microscopic images underwent enhancement, followed by conversion into binary black-and-white format. Subsequently, the boundary contours of the particle shapes were extracted. During image analysis and data processing, particles exhibited incomplete boundaries–only a partial outline was visible within the micrograph. The remainder of particles that extends beyond the image frame was excluded from consideration to minimize measurement errors.

## 3. Results

**Figure 1:**
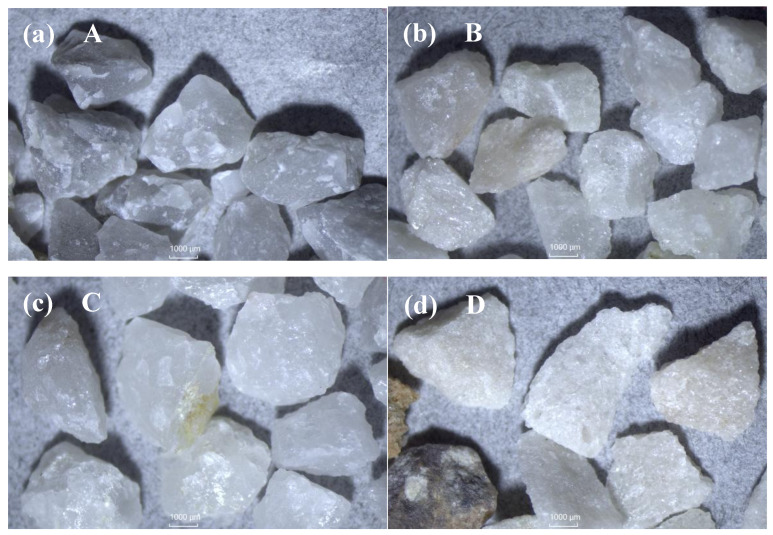
Images of quartz from different origins: (**a**) Xiangyang, (**b**) Fengyang, (**c**) Chengde and (**d**) Luoyang.

The optical microscope images of quartz particles are shown in Figure 1. The shapes of coarse quartz were similar. The color of quartz from Xiangyang tended to be grayish white, while other quartz was whiter except for some dark particles.

### 3.1. Particle Size

After employing an identical crushing and grinding procedure, the particle size and distribution of quartz particles varied according to the geographical origin. The particle size parameters of the quartz from four origins are listed in Table 2, Table 3, Table 4 and Table 5 (the frequency distribution is shown in Figures S1–S4). The diameter of an area equivalent projection circle (EQPC diameter, *d_e_*) was calculated as Equation (1), where the projected area of the particle is A.

*d_e_* = (4A/π)^1/2^ (1)

When the EQPC diameter is close to the D_50_ values, this indicates an approximate normal distribution. The average EQPC diameter and D_50_ of quartz from Xiangyang were the lowest for the 4–20 mesh particles. The minimum of the above values was observed for the 20–50 mesh and 70–100 mesh quartz from Luoyang. The 50–70 mesh quartz from Chengde had the lowest particle size. Skewness (SK) is an indicator of the symmetry of frequency distribution curve, indicating the degree of coarseness or fineness [18]. Folk & Ward skewness (*SK_I_*) formula is as follows [19]:

(2) $${SK}_{I}=\frac{\emptyset_{16}+\emptyset_{84}-2\emptyset_{50}}{2\left(\emptyset_{84}-\emptyset_{16}\right)}+\frac{\emptyset_{5}+\emptyset_{95}-2\emptyset_{50}}{2\left(\emptyset_{95}-\emptyset_{5}\right)}$$

where ∅ = −log_2_D.

As determined from *SK_I_* of the EQPC diameter, the particle size of most quartz particles exhibited a nearly symmetric distribution (*SK_I_* = −0.1 to +0.1). Negative skewness ranged from −0.1 to −0.3, representing a coarse-dominant distribution (indicating a tail of coarser particles), e.g., the particle size of 10–20 mesh quartz particles from Xiangyang, 8–10 mesh quartz from Chengde, and 20–50 mesh quartz from Luoyang. However, quartz particles with 6–8 mesh from Xiangyang presented a fine-skewed distribution (indicating an excess of fine particles) for skewness = 0.2. An *SK_I_* of higher than 0.3 corresponded to a very fine-skewed distribution, such as in quartz particles with 20–50 mesh from Fengyang. For the quality control, it is better to select particles with a symmetric distribution.

The ratio of (D_90_ − D_10_)/D_50_, referred to as the uniformity coefficient, is a critical parameter in particle size analysis. It primarily characterizes the breadth of the particle size distribution. A higher uniformity coefficient indicates a broader particle size distribution, whereas a lower uniformity coefficient corresponds to a narrower distribution. The particle size distribution of quartz from Fengyang exhibited a relatively smooth and regular profile, with the lowest uniformity coefficient observed in the coarse particle size range (e.g., 0.217 for 4–6 mesh). This suggests a concentrated particle size distribution, indicative of superior homogeneity and an effective crushing process. The uniformity coefficients for the 10–20 mesh and 20–50 mesh fractions were 0.755 and 0.784, respectively, reflecting a broad particle size distribution for quartz from Luoyang. Quartz particles from Xiangyang and Chengde exhibited intermediate characteristics in terms of particle-size distribution. However, a wide particle size range was evident in the medium particle size fraction, which likely significantly influenced particle packing behavior.

### 3.2. Flatness and Circularity

The flatness *e* is defined as the ratio of the maximum (F_max_) to the minimum (F_min_) Feret diameter in the projected shape of particles [20]:

*e* = F_max_/F_min_ (3)

The flatness data of quartz particles of varying sizes from four different regions were analyzed, where particle flatness was calculated according to Equation (3). The statistical results are presented in Table 6 (the frequency distribution is shown in Figures S5–S8). The flatness of all quartz was basically equivalent to the values (1.21–1.56) of the elongation index for quartz sands [21]. Considering regional differences, quartz D (Luoyang) exhibited the highest overall flatness values (e.g., 1.654 for the 10–20 mesh fraction), indicating particles with notably flat or elongated shapes. In contrast, quartz B (Fengyang) demonstrated the lowest overall flatness (e.g., 1.379 for the 20–50 mesh fraction), with particles exhibiting an approximate equiaxed morphology and good shape uniformity. The flatness values for quartz A (Xiangyang) and C (Chengde) fell between these two extremes. Regarding particle size variation, the flatness of quartz D (Luoyang) was higher in the coarse-grained range but decreased markedly in finer fractions, converging toward values observed in other regions. The flatness of quartz B (Fengyang) remained relatively stable across particle sizes. For quartz A (Xiangyang) and C (Chengde), the flatness peaked within the 10–70 mesh range. Notably, at the finest particle size range (70–100 mesh), flatness values converged (ranging from 1.467 to 1.491), suggesting that the morphology of very fine particles tends to be similar regardless of the origin.

The circularity *Cr* is expressed as the ratio between the circumference of a circle with the same projected area A as the particle and the actual perimeter P of the particle’s projected contour [7]:

*Cr* = 4πA/P^2^ (4)

If the *Cr* value of a particle approaches 1, the particle tends to have a perfectly circular shape; conversely, lower *Cr* values represent a more irregular morphology. This parameter effectively characterizes the overall morphological features of the particle.

The circularity of particles was calculated based on Equation (4). The circularity data of quartz particles are listed in Table 7 (the frequency distribution is shown in Figures S9–S12). Quartz B from Fengyang exhibited the highest and most consistent circularity values (ranging from 0.820 to 0.826). By contrast, quartz D (Luoyang) showed significantly lower circularity values (0.755 to 0.759) in the coarse to medium grain size range (6–20 mesh), indicating irregular particle shapes; however, the circularity markedly increased in the fine grain size range. Quartz from sites A (Xiangyang) and C (Chengde) displayed intermediate circularity values, with site A generally exhibiting slightly higher circularity than site C in the fine fraction. The circularity values of quartz A and D exhibited an overall increasing trend as particle size decreased. Generally, when circularity values generally exceeded 0.80, the particles presented a regular/circular shape. Notably, quartz from site D (Luoyang) demonstrated the most pronounced increase in circularity, rising from 0.755 for 4–6 mesh to 0.806 for 70–100 mesh, reflecting a significant improvement in particle shape regularity during fine crushing. Conversely, quartz from site B (Fengyang) maintained consistently high and unchanged circularity values throughout.

### 3.3. Angularity

The angularity *A_u_* is defined in Equation (5) [22]:

*A_u_* = (P_convex_/P_ellipse_)^2^ (5)

where P_convex_ denotes the convex perimeter, and P_ellipse_ denotes the perimeter of an equivalent ellipse. Larger values of angularity indicate that particles are closer to a spherical or ellipsoid shape.

The calculated angularity of quartz with varying sizes from four distinct regions is listed in Table 8 (the frequency distribution is shown in Figures S13–S16). Quartz from site B (Fengyang) exhibited the lowest angularity in the coarse grain size range (1.090 for 4–6 mesh), indicating the smoothest particle contours and the least pronounced angularity. Nevertheless, quartz from site D (Luoyang) demonstrated the highest overall angularity in the coarse to medium grain size stages (6–20 mesh), exemplified by a value of 1.135 for 4–6 mesh, representing more distinct angular features. The angularity values for quartz from sites A (Xiangyang) and C (Chengde) were intermediate and closely aligned between those of sites B and D. Examining the influence of particle size, quartz from all origins exhibited a decreasing trend in the angularity as particle size decreased. Notably, the angularity of quartz D (Luoyang) showed the most pronounced reduction, declining from 1.135 (4–6 mesh) to 1.075 (20–50 mesh), indicating a significant attenuation of particle angularity during fine grinding. Conversely, the angularity of quartz B (Fengyang) slightly increased to 1.098 within the 20–50 mesh range. Similar phenomena can be seen in the variation in the angularity for quartz C and D of 70–100 mesh.

### 3.4. Fractal Dimension

By linear fitting the data points of perimeter *P* and area *A* on the double logarithmic coordinates, the fractal dimension *D* is derived to be twice the slope [23]:

lg*P* = (*D*/2)lg*A* + *k*_0_ (6)

where *k*_0_ represents a constant.

**Figure 2:**
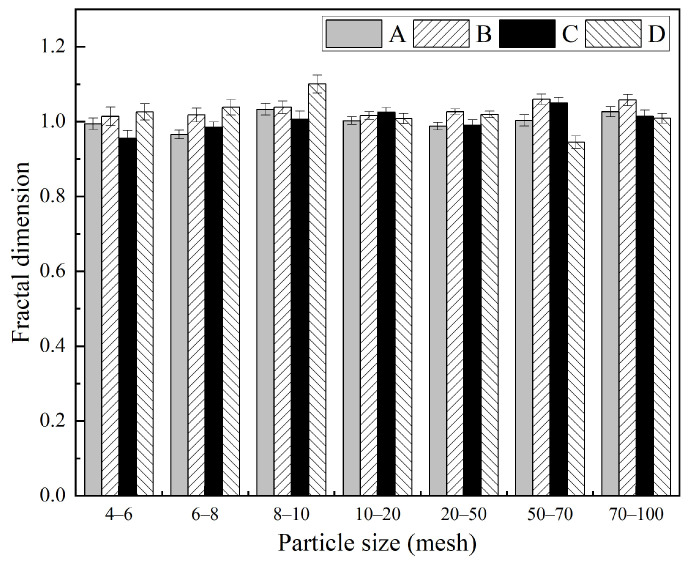
Fractal dimensions of quartz particles from different origins.

The fractal dimensions *D* of quartz particles with seven distinct particle size fractions from four different regions were determined employing the area–perimeter method. The fitting diagrams illustrating the relationship between area and perimeter for quartz particles are shown in Figures S17–S20. The fractal dimensions corresponding to quartz particles across the different particle sizes and origins indicated that the particle shape was statistically self-similar. Overall, the fractal dimension of quartz was in inverse proportion to the particle size except for quartz D, as seen in Figure 2. Accompanied by fragmentation and refinement, the particles of quartz became more complex [24]. From a geographical perspective, 20–100 mesh quartz B (Fengyang) particles exhibited the highest fractal dimensions, ranging from 1.027 to 1.060. Such a small change was attributed to the rough outline, despite the low flatness and high circularity of the particles. Conversely, quartz D (Luoyang) particles demonstrated a notably high fractal dimension (up to 1.100) in the coarse grain size range (e.g., 8–10 mesh), albeit with significant fluctuations. This variability was explained by the high flatness and low circularity observed in coarse particles from this region. Quartz particles from origins A (Xiangyang) and C (Chengde) generally exhibited lower fractal dimensions, predominantly between 0.95 and 1.05, reflecting moderate flatness and circularity. Furthermore, the goodness-of-fit values (R^2^) are consistently above 0.85, with most exceeding 0.90, and reaching as high as 0.988 for 20–50 mesh size in region B. These results indicated that the fractal dimension effectively characterizes particle morphology and that the data are highly reliable.

## 4. Discussion

The morphology of quartz particles varied due to the combined influence of their geological origins [25] and the crushing and grinding processes. Specifically, quartz from Fengyang and Luoyang predominantly originated from sedimentary formations such as quartz sandstone and siliceous rock. In contrast, quartz from Xiangyang and Chengde is primarily derived from sedimentary metamorphic deposits and quartzite, respectively. Notably, quartz from Luoyang exhibited the highest flatness and a pronounced angularity, which may be attributed to the preservation of edges and corners during sedimentation, diagenesis, and crushing processes, resulting in particles that are predominantly flat and narrow. Quartz from Fengyang had the highest circularity alongside the lowest angularity, likely due to its granular ore structure characterized by uniform geological formation and slow diagenesis–Fengyang’s quartz deposit, formed during the Proterozoic era, represents the oldest quartzite deposit in China. For quartz from Xiangyang and Chengde, the difference in the grain size resulted in a clear contrast for particle sizes [26]. Furthermore, as particle size decreased, the shape characteristics across all regions converged, suggesting that the influence of the original geological structure diminished during fine crushing, with the crushing mechanism becoming the dominant factor shaping particle morphology.

The Pearson correlation coefficient (R), a measurement quantifying the strength of the association between two variables, can be calculated as follows [27]:

*R_X_*_,*Y*_ = cov(X,Y)/σ_X_σ_Y_ (7)

where the function cov denotes the covariance of variables X and Y, and σ_X_ and σ_Y_ denote the standard deviation of variables X and Y, respectively.

**Figure 3:**
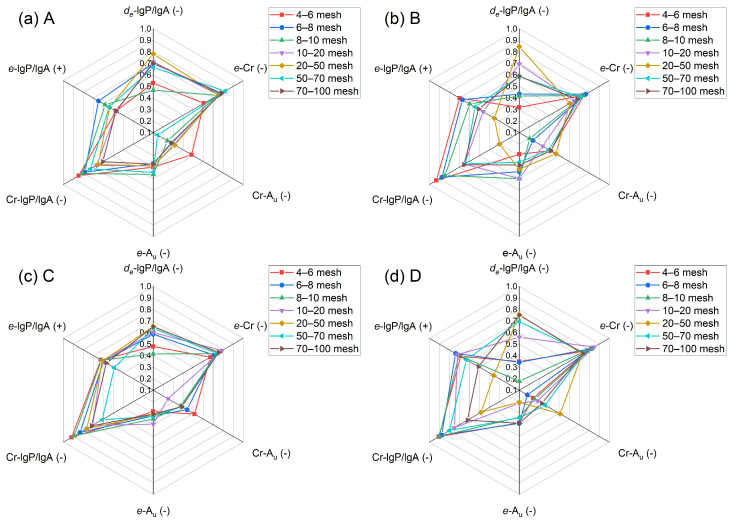
Pearson correlation coefficients of the size and shape parameters for quartz with different sizes.

The correlation of the size and shape parameters for quartz from different origins was evaluated by calculating Pearson coefficients as listed in Table 9, Table 10, Table 11 and Table 12. The flatness presented the strongest negative correlation with circularity. The dimensionless ratio of lgP and lgA (Log of perimeter/Log of area) can be used in the global analysis of regularity of particle shape. For quartz from Xiangyang and Luoyang, the correlation degree between lgP/lgA and EQPC diameter was obvious (|R| ≥ 0.70). Moreover, the highest correlation between circularity and lgP/lgA could be found in quartz from Chengde (Table 11). Angularity can be regarded as an independent parameter due to the low Pearson correlation coefficient with others. Although the characteristics of quartz could not be identified by Pearson correlation coefficient alone [28], the comparison with the correlation between size and shape parameters may have provided some valuable information for reference. From Pearson correlation coefficients of the size and shape parameters for quartz with different sizes (Figure 3), quartz particles of 4–6 mesh from Xiangyang and Chengde were similar in the correlation, particularly circularity and angularity. Quartz particles of 20–50 mesh from Fengyang and Luoyang exhibited different correlations than other quartz particles. Therefore, attention should be given to the processing technology applied to the quartz particles in this specific range.

## 5. Conclusions

This study presents the results of a quantitative analysis of the shape parameters of quartz particles sourced from Xiangyang (Hubei), Fengyang (Anhui), Chengde (Hebei), and Luoyang (Henan) using a digital imaging process. The main conclusions are as follows:

(1) Due to the discrepancy in hardness and grain size, quartz from various origins presented different particle size distributions, even after using the same crushing and grinding processes. In general, the particle sizes of 4–20 mesh quartz from Xiangyang were the smallest. The highest particle sizes corresponded to 4–10 mesh quartz from Luoyang.

(2) Quartz particles from Luoyang exhibit the highest flatness values, coupled with low circularity in coarse to medium sizes, reflecting irregular shapes characterized by notably flat or elongated particles and poor uniformity. Conversely, quartz from Fengyang has the lowest flatness and the highest, most stable circularity, with particles exhibiting equiaxed forms and good shape uniformity. Quartz particles from Xiangyang and Chengde present intermediate characteristics between the above two origins. Furthermore, as particle size decreases, the flatness and circularity metrics across all regions converge (flatness 1.467–1.491 and circularity 0.806–0.827 for particles of 70–100 mesh), suggesting the homogenization of particle shape in finer fractions.

(3) Regarding outline morphology, quartz from Fengyang exhibits the lowest angularity, indicating smooth particle profiles. In contrast, quartz from Luoyang shows generally high angularity at coarse and medium sizes, reflecting pronounced edges and corners. The angularity of quartz from Xiangyang and Chengde is comparable and falls between the aforementioned values. Additionally, the fractal dimension of quartz particles ranges from 0.95 to 1.10, confirming their self-similar nature. Quartz particles of 20–100 mesh from Fengyang have rougher outlines considering the highest fractal dimensions.

(4) The geometric morphology of quartz particles depends on their geographic origin. The differences in quartz particles can be accurately determined through the comparison of the correlation of size and shape parameters. According to Pearson correlation coefficients with significant differences, an optimal strategy should be developed for processing applied to quartz particles of 20–50 mesh from Fengyang and Luoyang. Statistical analyses and quantification of particle characteristics provide a foundational framework for optimizing the selection of raw materials and for conducting quality control during the production of silica-based dry ramming mixes.

## Figures and Tables

**Table 1:** Chemical compositions of quartz.

Origin	SiO_2_	Al_2_O_3_	Fe_2_O_3_	CaO	MgO	K_2_O	Na_2_O	TiO_2_	I.L
A	97.81	0.24	0.60	0.328	0.298	0.045	0.023	0.027	0.63
B	98.49	0.60	0.45	0.024	0.086	0.121	0.012	0.021	0.19
C	98.31	0.57	0.57	0.023	0.058	0.120	0.015	0.075	0.26
D	98.55	0.29	0.59	0.030	0.075	0.090	0.011	0.065	0.29

**Table 2:** Particle size parameters of quartz from Xiangyang, Hubei.

Size	Average EQPC Diameter (mm)	D_10_ (mm)	D_50_ (mm)	D_90_ (mm)	SK_I_	(D_90_ − D_10_)/D_50_
4–6 mesh	4.646 ± 0.583	3.935	4.614	5.419	−0.022	0.322
6–8 mesh	2.968 ± 0.596	2.211	3.004	3.709	0.204	0.499
8–10 mesh	2.586 ± 0.366	2.159	2.571	3.032	0.053	0.340
10–20 mesh	1.354 ± 0.373	0.960	1.259	1.906	−0.24	0.751
20–50 mesh	0.728 ± 0.182	0.495	0.725	0.974	0.099	0.661
50–70 mesh	0.323 ± 0.051	0.258	0.321	0.388	0.066	0.406
70–100 mesh	0.216 ± 0.035	0.176	0.210	0.262	−0.076	0.410

**Table 3:** Particle size parameters of quartz from Fengyang, Anhui.

Size	Average EQPC Diameter (mm)	D_10_ (mm)	D_50_ (mm)	D_90_ (mm)	SK_I_	(D_90_ − D_10_)/D_50_
4–6 mesh	5.338 ± 0.428	4.731	5.345	5.891	0.063	0.217
6–8 mesh	3.097 ± 0.332	2.676	3.086	3.554	0.004	0.285
8–10 mesh	2.680 ± 0.285	2.306	2.690	3.018	0.073	0.265
10–20 mesh	1.502 ± 0.225	1.225	1.492	1.791	0.085	0.380
20–50 mesh	0.666 ± 0.169	0.423	0.683	0.858	0.33	0.638
50–70 mesh	0.343 ± 0.046	0.289	0.339	0.395	−0.057	0.311
70–100 mesh	0.263 ± 0.034	0.222	0.261	0.304	−0.022	0.315

**Table 4:** Particle size parameters of quartz from Chengde, Hebei.

Size	Average EQPC Diameter (mm)	D_10_ (mm)	D_50_ (mm)	D_90_ (mm)	SK_I_	(D_90_ − D_10_)/D_50_
4–6 mesh	5.107 ± 0.500	4.424	5.116	5.717	0.093	0.253
6–8 mesh	3.591 ± 0.512	2.927	3.578	4.299	0.067	0.384
8–10 mesh	2.717 ± 0.308	2.365	2.681	3.139	−0.118	0.289
10–20 mesh	1.827 ± 0.369	1.385	1.769	2.333	−0.067	0.536
20–50 mesh	0.839 ± 0.150	0.641	0.833	1.027	0.095	0.463
50–70 mesh	0.264 ± 0.049	0.203	0.258	0.332	−0.030	0.499
70–100 mesh	0.217 ± 0.034	0.173	0.215	0.259	0.053	0.401

**Table 5:** Particle size parameters of quartz from Luoyang, Henan.

Size	Average EQPC Diameter (mm)	D_10_ (mm)	D_50_ (mm)	D_90_ (mm)	SK_I_	(D_90_ − D_10_)/D_50_
4–6 mesh	5.129 ± 0.655	4.300	5.055	5.977	−0.065	0.332
6–8 mesh	3.929 ± 0.611	3.144	3.904	4.722	0.059	0.404
8–10 mesh	2.955 ± 0.382	2.462	2.941	3.494	0.017	0.351
10–20 mesh	1.738 ± 0.489	1.158	1.695	2.438	0.032	0.755
20–50 mesh	0.464 ± 0.148	0.321	0.434	0.661	−0.138	0.784
50–70 mesh	0.297 ± 0.041	0.244	0.296	0.347	0.045	0.348
70–100 mesh	0.194 ± 0.034	0.156	0.193	0.239	0.050	0.428

**Table 6:** Flatness *e* of quartz particles.

Size	A	B	C	D
4–6 mesh	1.440 ± 0.217	1.396 ± 0.231	1.396 ± 0.226	1.552 ± 0.368
6–8 mesh	1.496 ± 0.339	1.413 ± 0.255	1.433 ± 0.240	1.630 ± 0.456
8–10 mesh	1.519 ± 0.298	1.437 ± 0.248	1.483 ± 0.281	1.628 ± 0.411
10–20 mesh	1.561 ± 0.334	1.398 ± 0.201	1.532 ± 0.310	1.654 ± 0.523
20–50 mesh	1.561 ± 0.319	1.379 ± 0.216	1.504 ± 0.309	1.466 ± 0.280
50–70 mesh	1.562 ± 0.328	1.472 ± 0.219	1.540 ± 0.318	1.466 ± 0.298
70–100 mesh	1.484 ± 0.259	1.481 ± 0.234	1.491 ± 0.309	1.467 ± 0.306

**Table 7:** Circularity *Cr* of quartz particles.

Size	A	B	C	D
4–6 mesh	0.792 ± 0.052	0.820 ± 0.054	0.815 ± 0.057	0.755 ± 0.065
6–8 mesh	0.789 ± 0.065	0.824 ± 0.053	0.805 ± 0.057	0.747 ± 0.077
8–10 mesh	0.789 ± 0.059	0.826 ± 0.051	0.800 ± 0.061	0.759 ± 0.077
10–20 mesh	0.787 ± 0.068	0.823 ± 0.044	0.795 ± 0.063	0.757 ± 0.092
20–50 mesh	0.794 ± 0.068	0.818 ± 0.052	0.806 ± 0.069	0.817 ± 0.070
50–70 mesh	0.809 ± 0.066	0.826 ± 0.052	0.798 ± 0.070	0.814 ± 0.064
70–100 mesh	0.827 ± 0.057	0.822 ± 0.056	0.805 ± 0.069	0.806 ± 0.064

**Table 8:** Angularity *A_u_* of quartz particles.

Size	A	B	C	D
4–6 mesh	1.115 ± 0.066	1.090 ± 0.054	1.098 ± 0.063	1.135 ± 0.069
6–8 mesh	1.103 ± 0.066	1.080 ± 0.054	1.098 ± 0.060	1.126 ± 0.079
8–10 mesh	1.095 ± 0.065	1.069 ± 0.053	1.091 ± 0.066	1.107 ± 0.076
10–20 mesh	1.086 ± 0.072	1.085 ± 0.053	1.083 ± 0.063	1.107 ± 0.078
20–50 mesh	1.075 ± 0.071	1.098 ± 0.064	1.077 ± 0.071	1.075 ± 0.070
50–70 mesh	1.054 ± 0.060	1.058 ± 0.053	1.077 ± 0.073	1.078 ± 0.066
70–100 mesh	1.054 ± 0.052	1.060 ± 0.060	1.083 ± 0.068	1.089 ± 0.068

**Table 9:** Pearson correlation coefficients of the size and shape parameters for quartz from Xiangyang, Hubei.

Pearson Correlation Coefficient *R*	*e*	*Cr*	*A_u_*	lg*P*/lg*A*	*d_e_*
**EQPC diameter, *d_e_***	0.100	−0.131	0.050	−0.700	1
**lg*P*/lg*A***	0.467	−0.605	0.167	1	
**Angularity, *A_u_***	−0.373	−0.280	1		
**Circularity, *Cr***	−0.784	1			
**Flatness, *e***	1				

**Table 10:** Pearson correlation coefficients of the size and shape parameters for quartz from Fengyang, Anhui.

Pearson Correlation Coefficient *R*	*e*	*Cr*	*A_u_*	lg*P*/lg*A*	*d_e_*
**EQPC diameter, *d_e_***	0.063	−0.216	0.192	−0.587	1
**lg*P*/lg*A***	0.506	−0.655	0.200	1	
**Angularity, *A_u_***	−0.381	−0.416	1		
**Circularity, *Cr***	−0.679	1			
**Flatness, *e***	1				

**Table 11:** Pearson correlation coefficients of the size and shape parameters for quartz from Chengde, Hebei.

Pearson Correlation Coefficient *R*	*e*	*Cr*	*A_u_*	lg*P*/lg*A*	*d_e_*
**EQPC diameter, *d_e_***	0.017	−0.051	0.053	−0.650	1
**lg*P*/lg*A***	0.572	−0.717	0.241	1	
**Angularity, *A_u_***	−0.306	−0.385	1		
**Circularity, *Cr***	−0.756	1			
**Flatness, *e***	1				

**Table 12:** Pearson correlation coefficients of the size and shape parameters for quartz from Luoyang, Henan.

Pearson Correlation Coefficient *R*	*e*	*Cr*	*A_u_*	lg*P*/lg*A*	*d_e_*
**EQPC diameter, *d_e_***	−0.023	−0.026	0.075	−0.750	1
**lg*P*/lg*A***	0.510	−0.619	0.133	1	
**Angularity, *A_u_***	−0.386	−0.326	1		
**Circularity, *Cr***	−0.739	1			
**Flatness, *e***	1				

## Data Availability

The original contributions presented in the study are included in the article/Supplementary Materials. Further inquiries can be directed to the corresponding author.

## Supplementary Materials

The following supporting information can be downloaded at: https://www.mdpi.com/article/10.3390/ma19061068/s1, Figure S1: Frequency distribution of quartz particles from Xiangyang, Hubei; Figure S2: Frequency distribution of quartz particles from Fengyang, Anhui; Figure S3: Frequency distribution of quartz particles from Chengde, Hebei; Figure S4: Frequency distribution of quartz particles from Luoyang, Henan; Figure S5: Flatness of quartz particles from Xiangyang, Hubei; Figure S6: Flatness of quartz particles from Fengyang, Anhui; Figure S7: Flatness of quartz particles from Chengde, Hebei; Figure S8: Flatness of quartz particles from Luoyang, Henan; Figure S9: Circularity of quartz particles from Xiangyang, Hubei; Figure S10: Circularity of quartz particles from Fengyang, Anhui; Figure S11: Circularity of quartz particles from Chengde, Hebei; Figure S12: Circularity of quartz particles from Luoyang, Henan; Figure S13: Angularity of quartz particles from Xiangyang, Hubei; Figure S14: Angularity of quartz particles from Fengyang, Anhui; Figure S15: Angularity of quartz particles from Chengde, Hebei; Figure S16: Angularity of quartz particles from Luoyang, Henan; Figure S17: Fitting curves of area (A) and perimeter (P) for quartz particles from Xiangyang, Hubei; Figure S18: Fitting curves of area (A) and perimeter (P) for quartz particles from Fengyang, Anhui; Figure S19: Fitting curves of area (A) and perimeter (P) for quartz particles from Chengde, Hebei; Figure S20: Fitting curves of area (A) and perimeter (P) for quartz particles from Luoyang, Henan.
