# Supplementary material for: Quantitative Analysis of the Shape Characteristics for Quartz Particles
Source: Materials (Basel). 2026 Mar 11;19(6):1068. doi: 10.3390/ma19061068 (PMC13028081; doi:10.3390/ma19061068)
Supplement: Supplementary file 1 [file materials-19-01068-s001.zip › materials-4163423-supplementary.pdf]

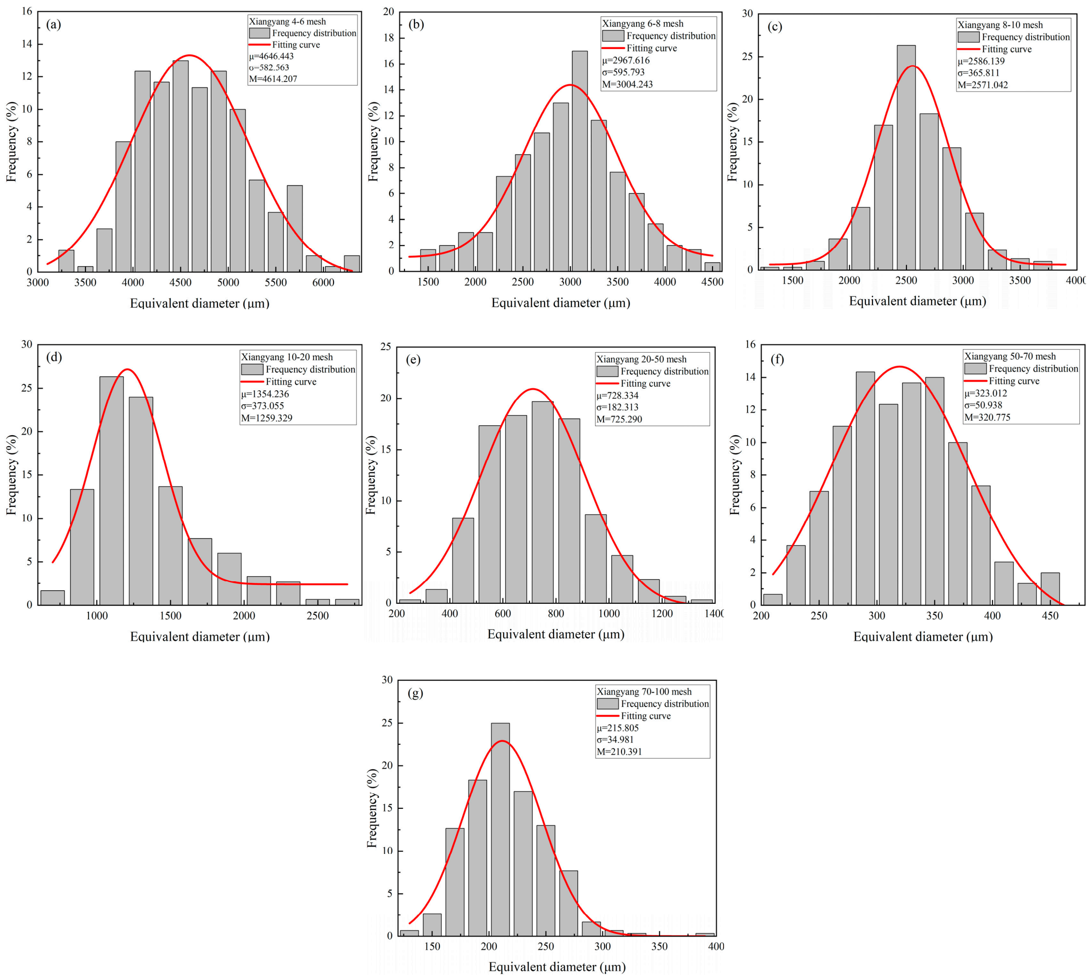

Figure S1. Frequency distribution of quartz particles from Xiangyang, Hubei.

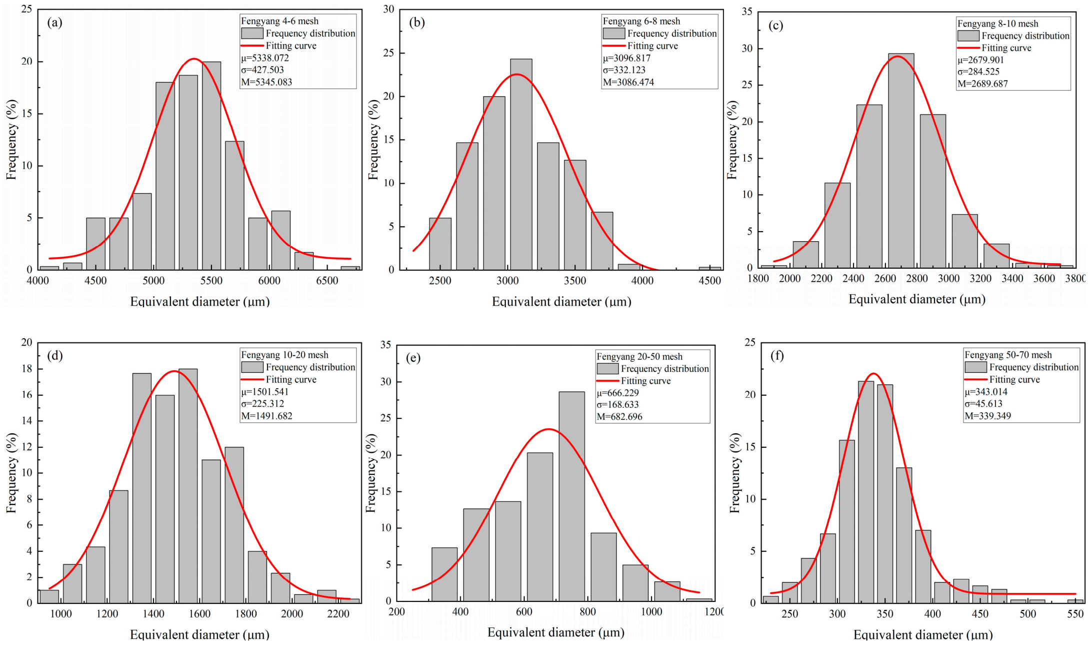

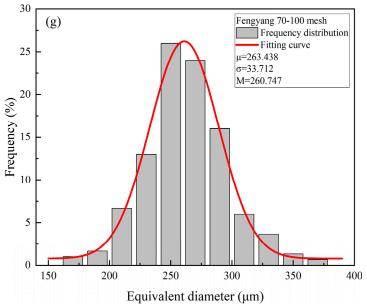

Figure S2. Frequency distribution of quartz particles from Fengyang, Anhui.

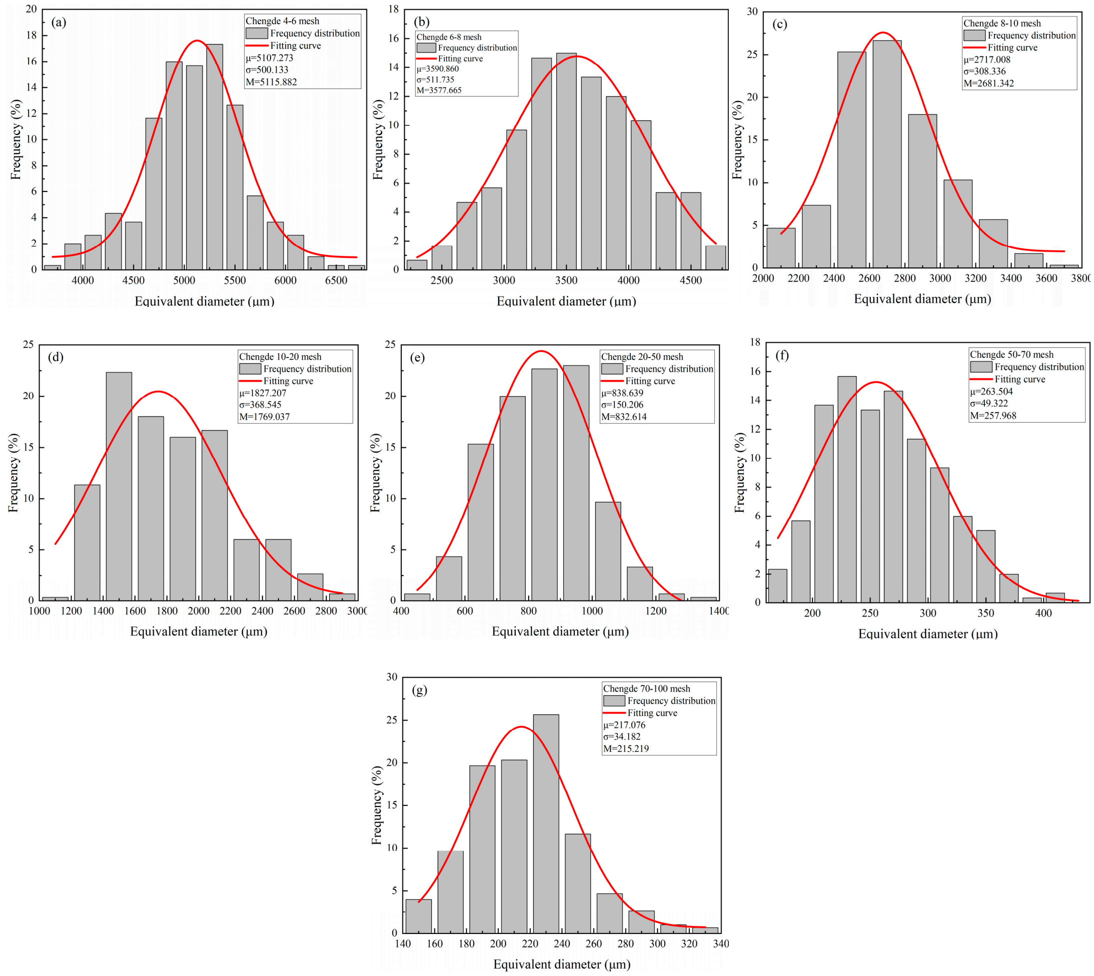

Figure S3. Frequency distribution of quartz particles from Chengde, Hebei.

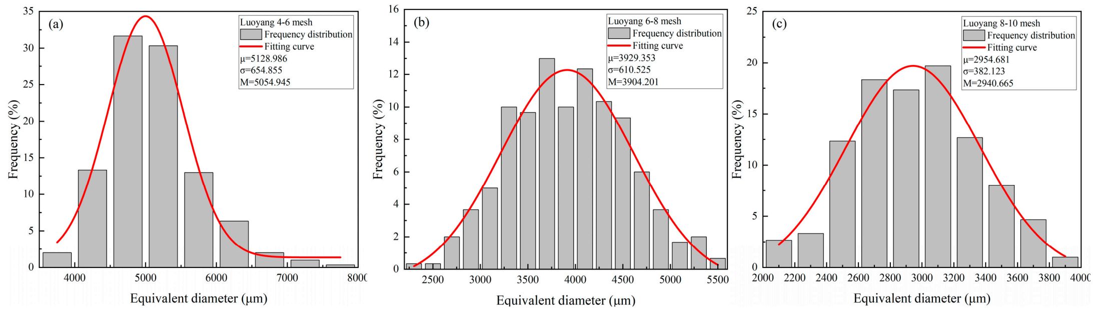

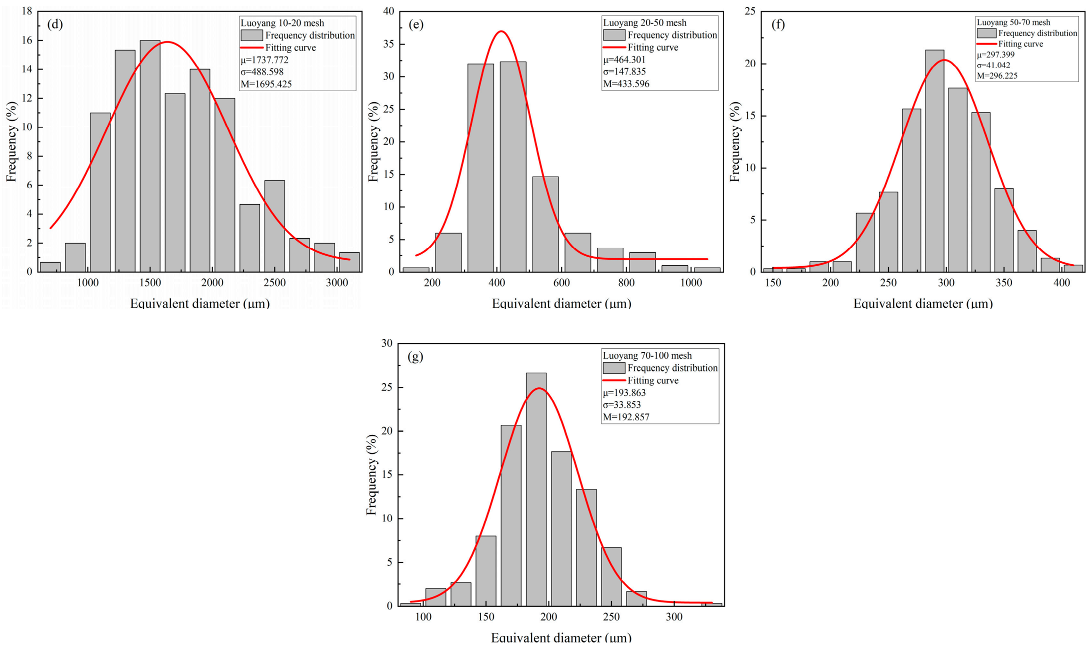

Figure S4. Frequency distribution of quartz particles from Luoyang, Henan.

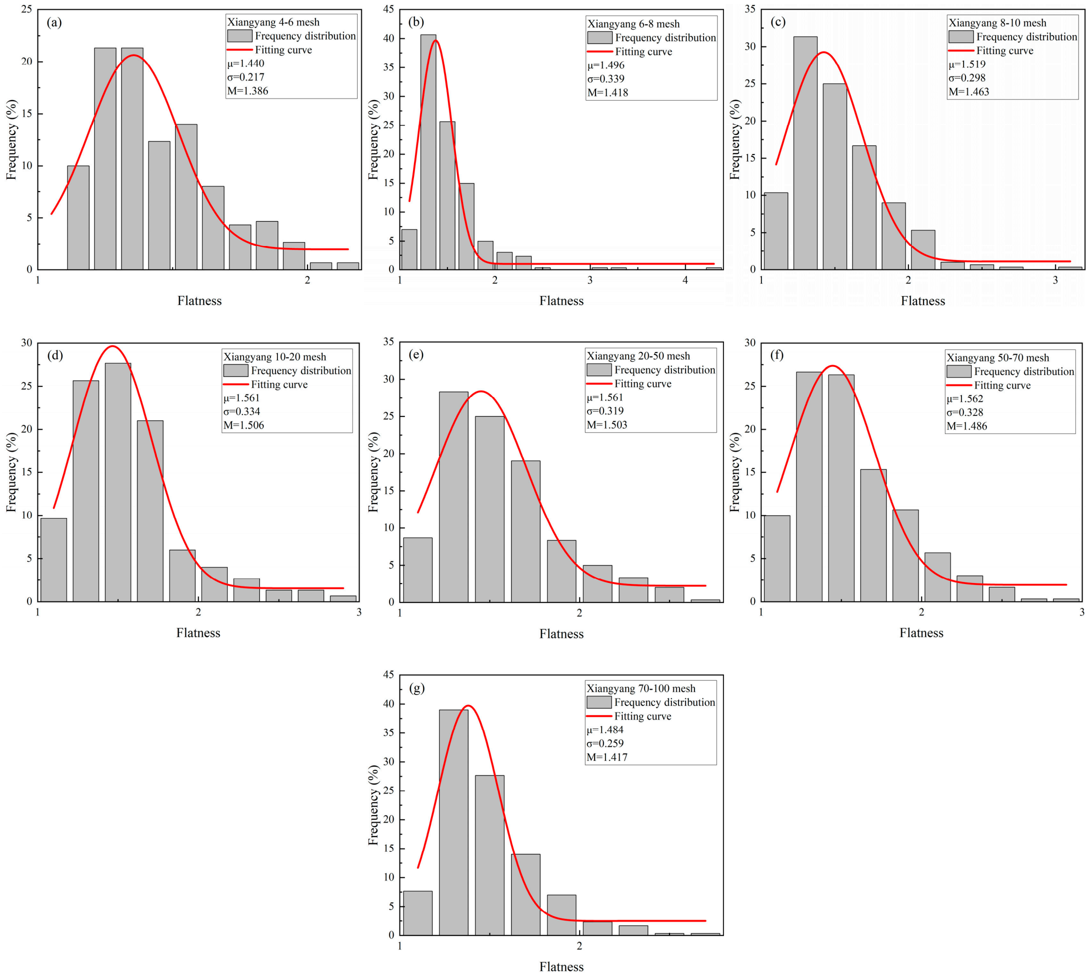

Figure S5. Flatness of quartz particles from Xiangyang, Hubei.

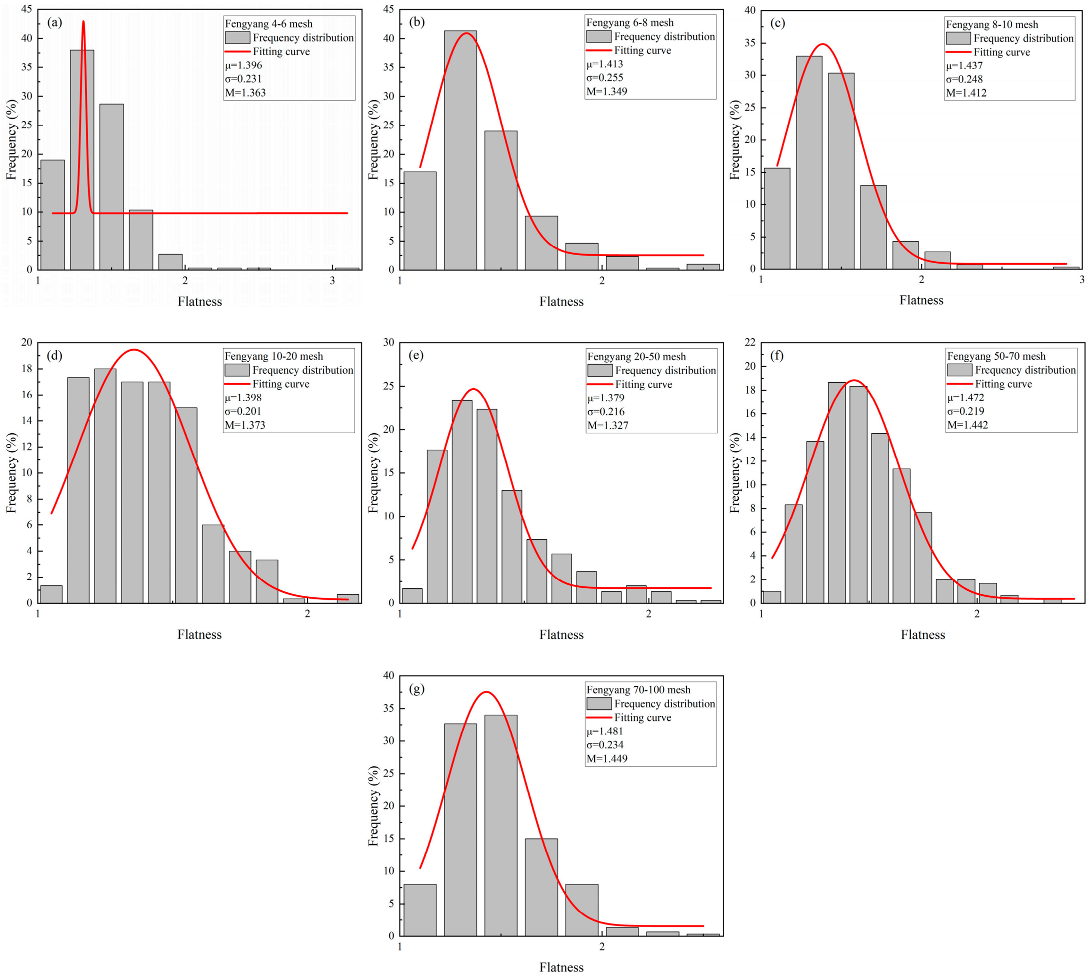

Figure S6. Flatness of quartz particles from Fengyang, Anhui.

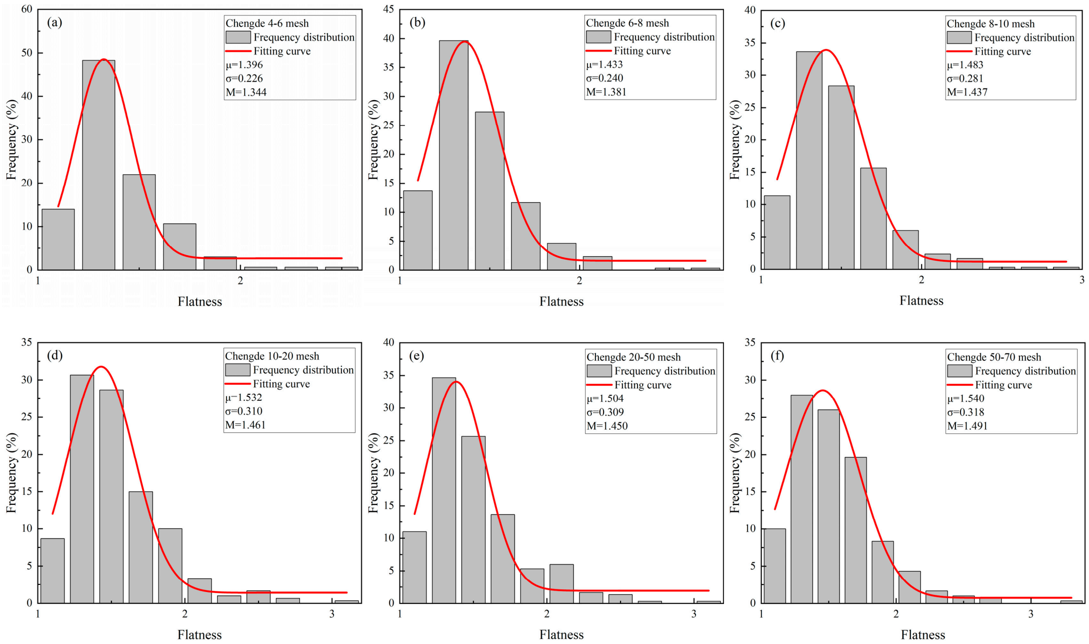

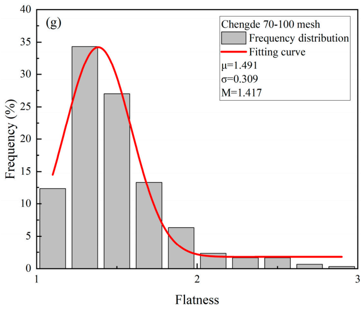

Figure S7. Flatness of quartz particles from Chengde, Hebei.

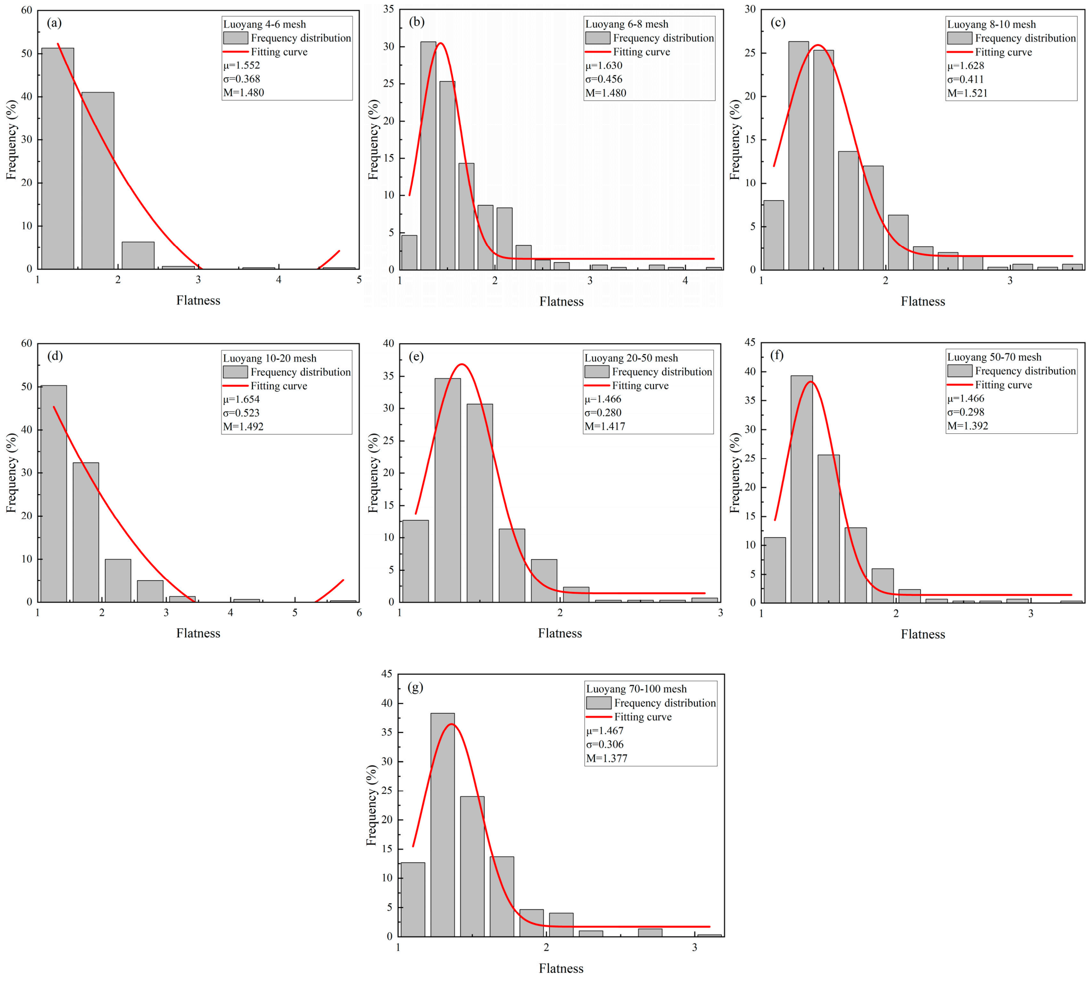

Figure S8. Flatness of quartz particles from Luoyang, Henan.

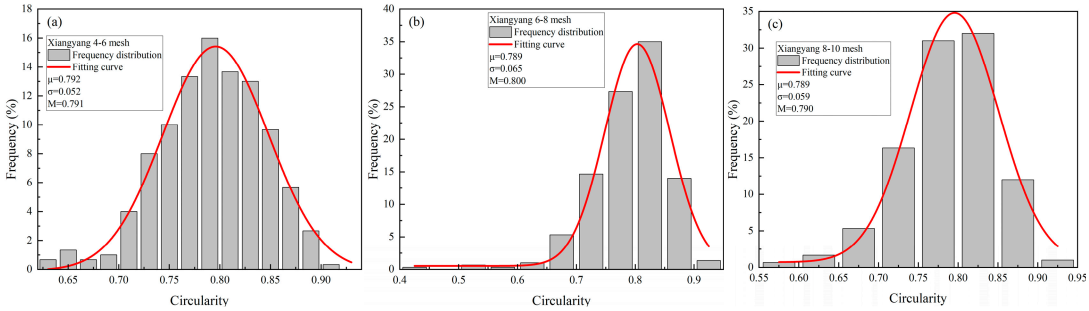

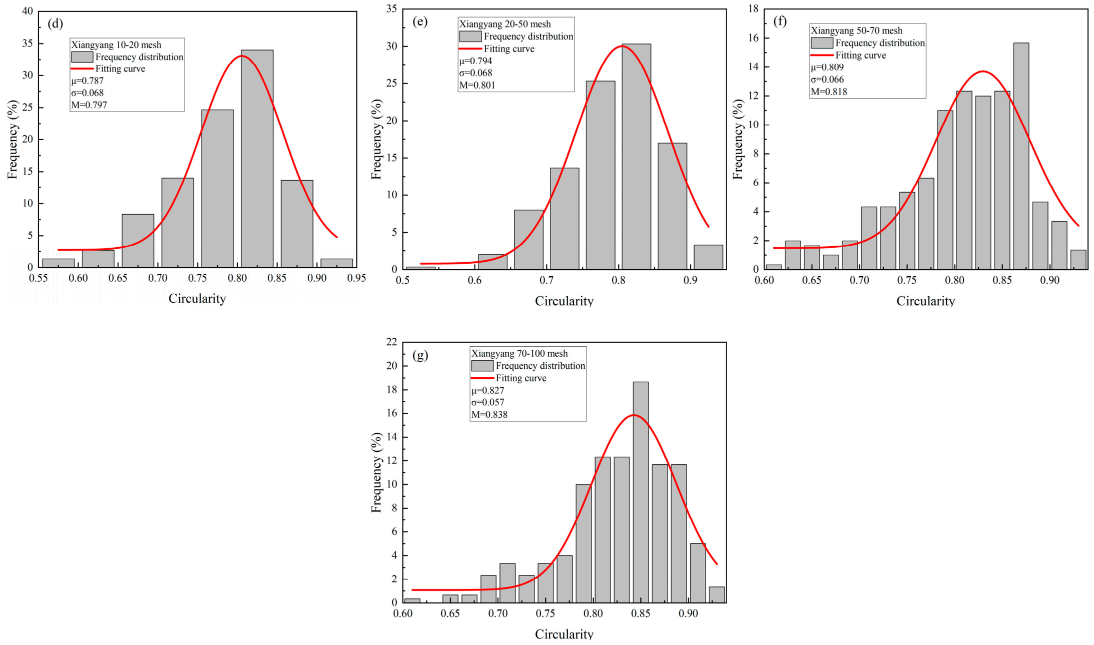

Figure S9. Circularity of quartz particles from Xiangyang, Hubei.

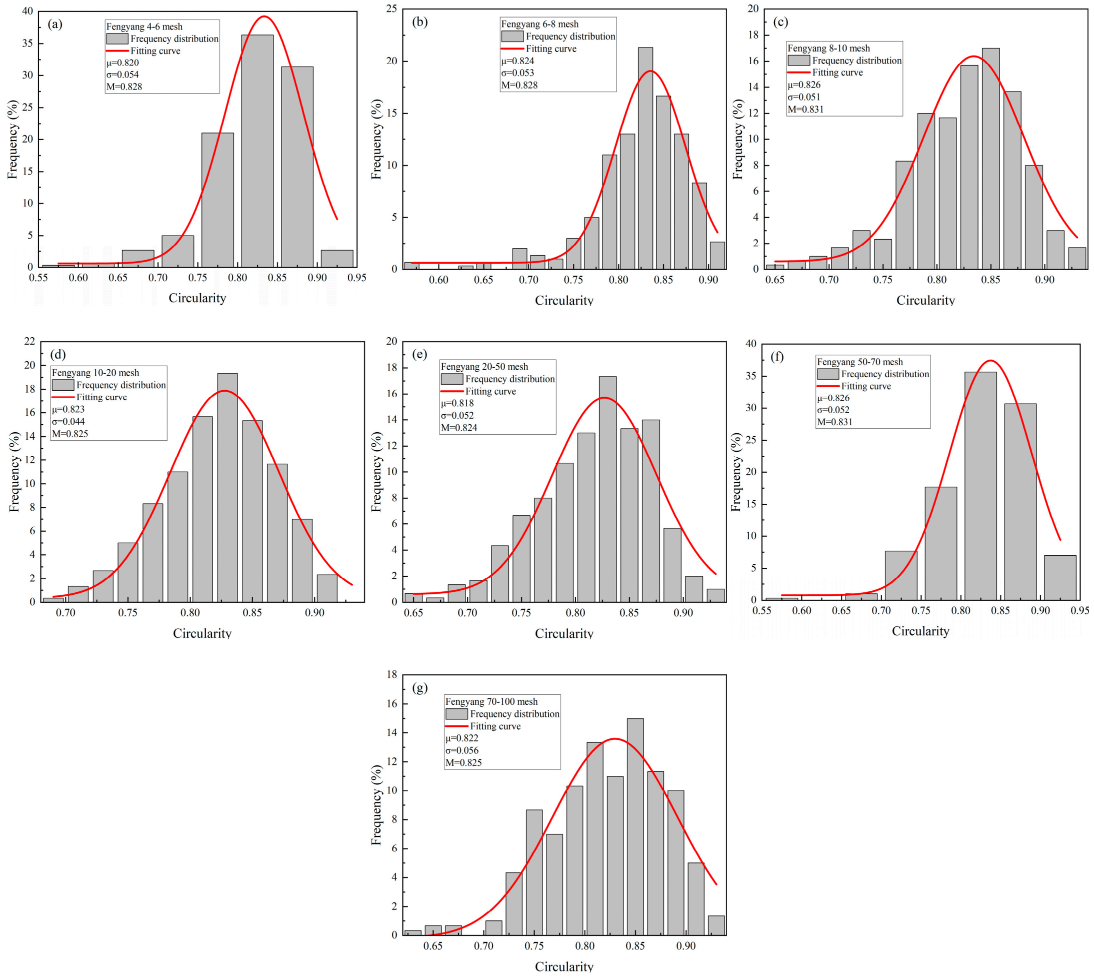

Figure S10. Circularity of quartz particles from Fengyang, Anhui.

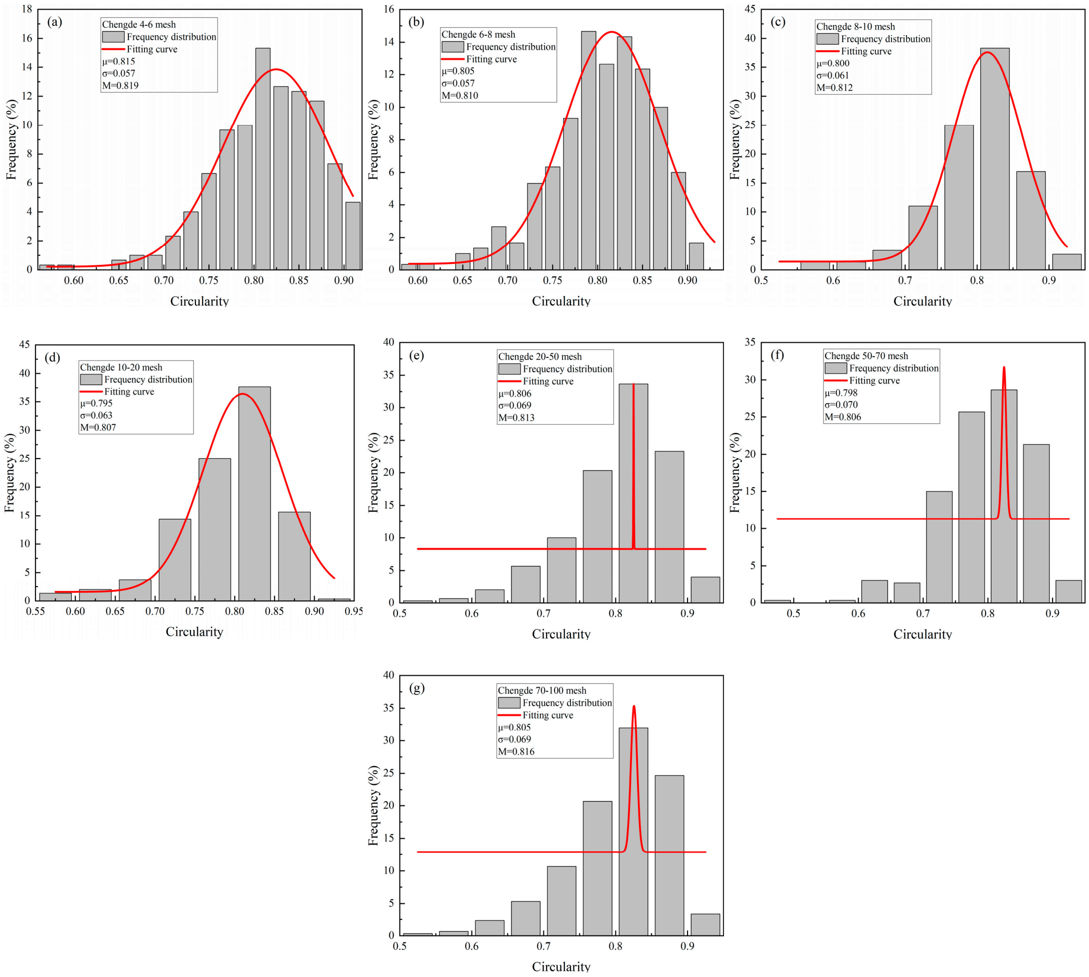

Figure S11. Circularity of quartz particles from Chengde, Hebei.

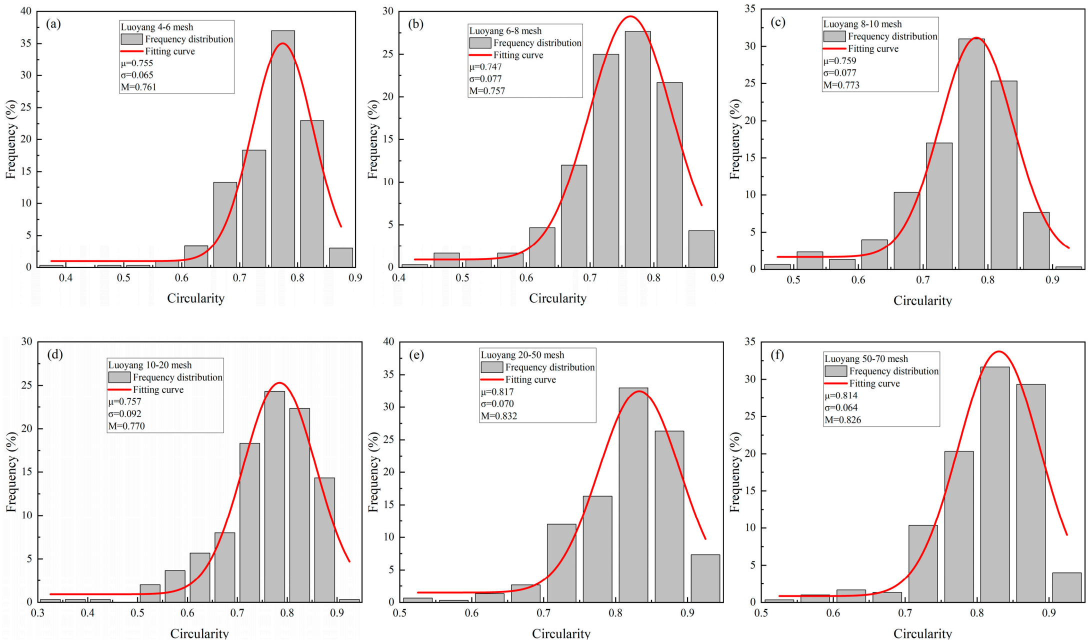

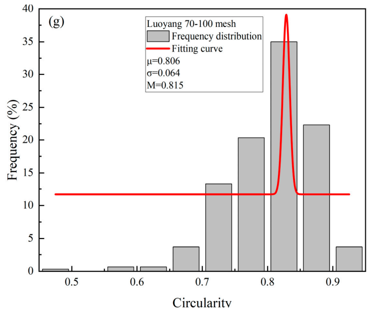

Figure S12. Circularity of quartz particles from Luoyang, Henan.

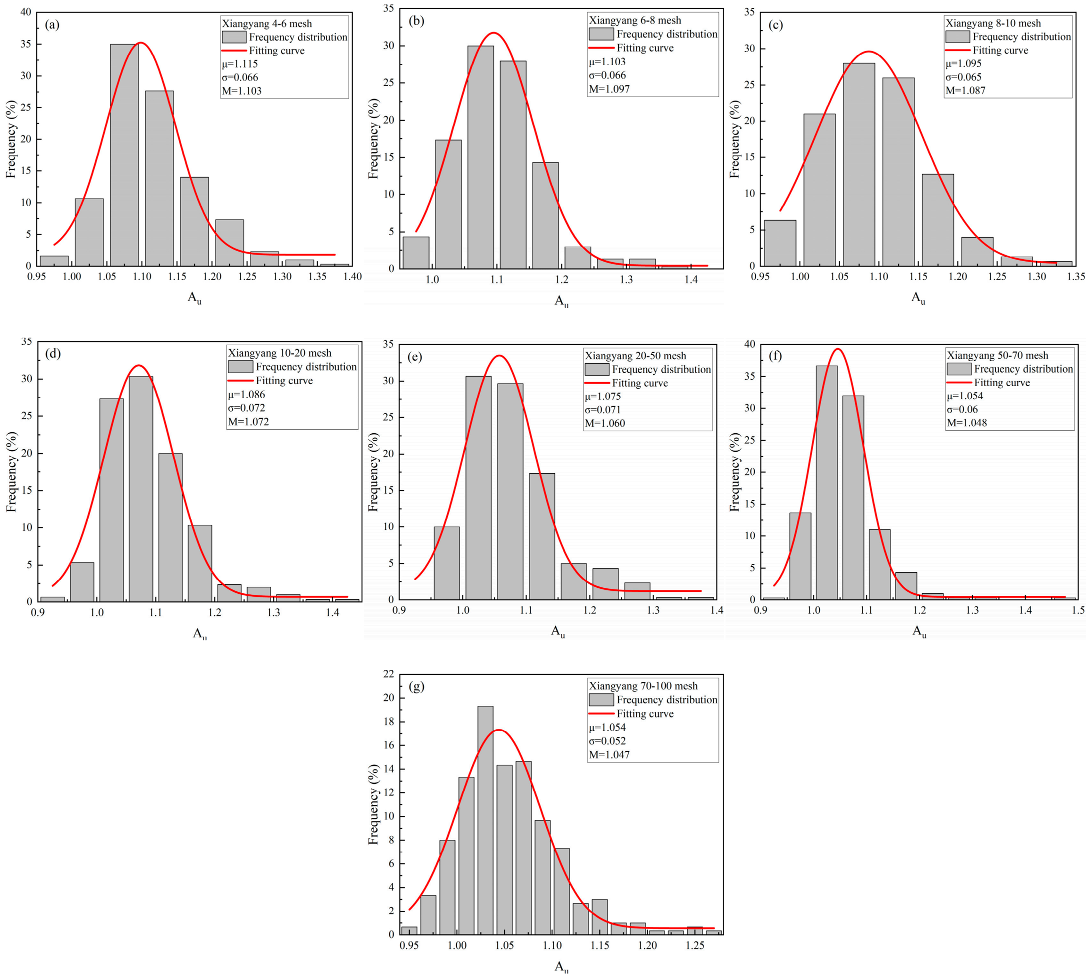

Figure S13. Angularity of quartz particles from Xiangyang, Hubei.

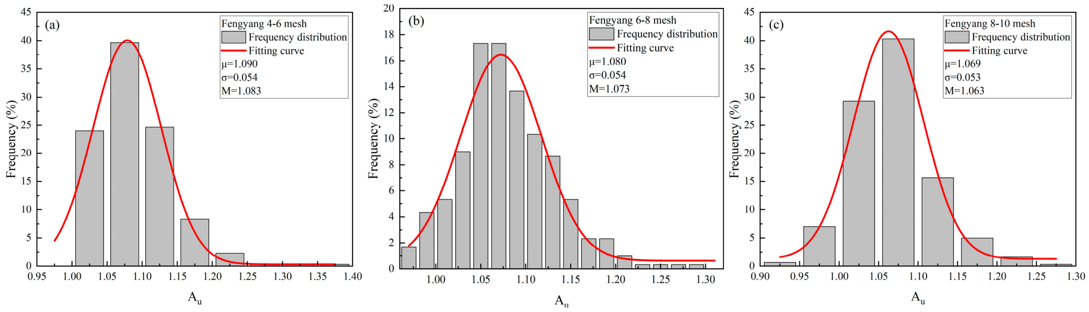

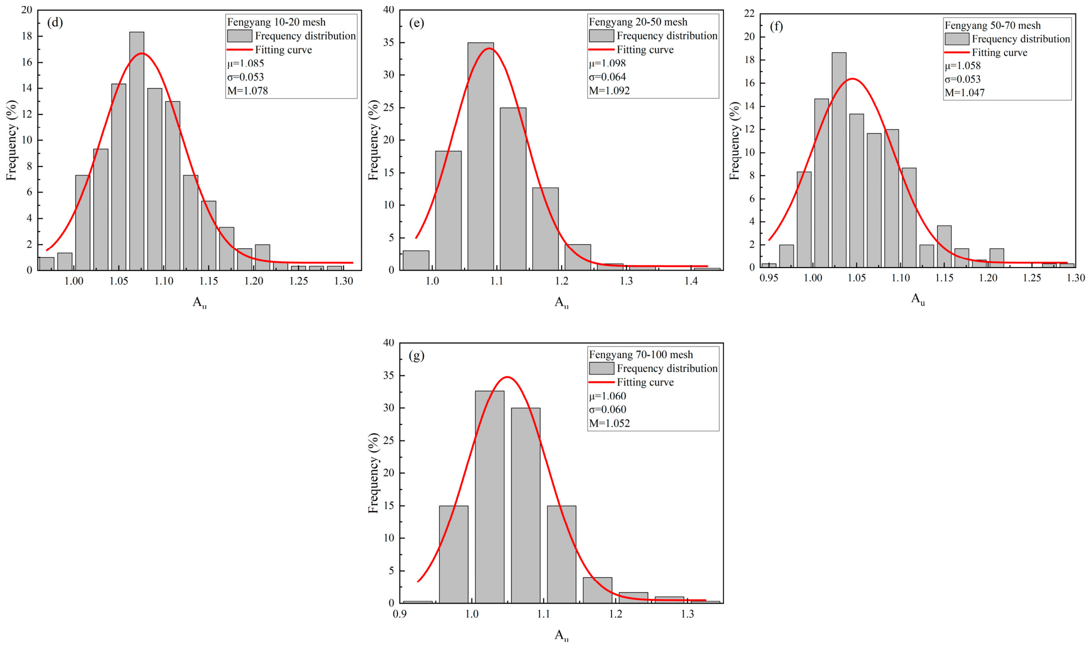

Figure S14. Angularity of quartz particles from Fengyang, Anhui.

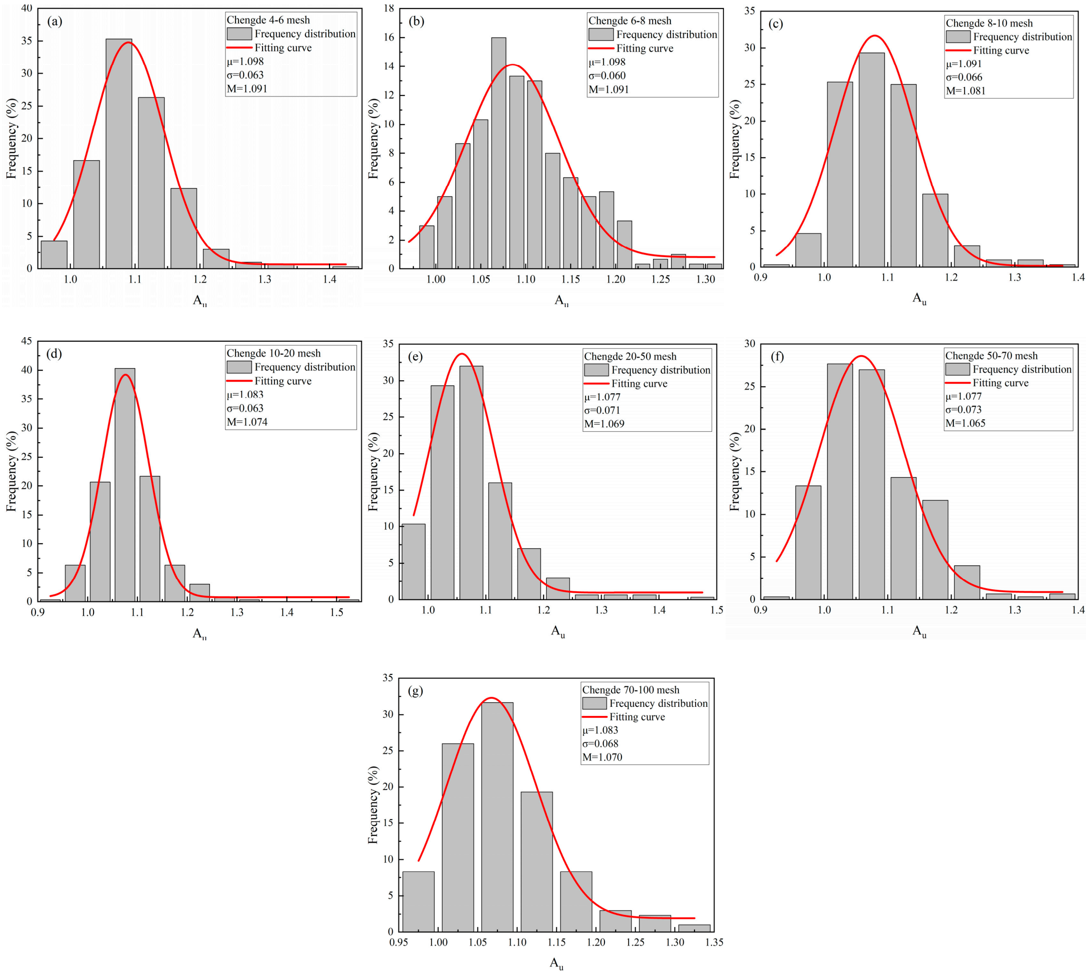

Figure S15. Angularity of quartz particles from Chengde, Hebei.

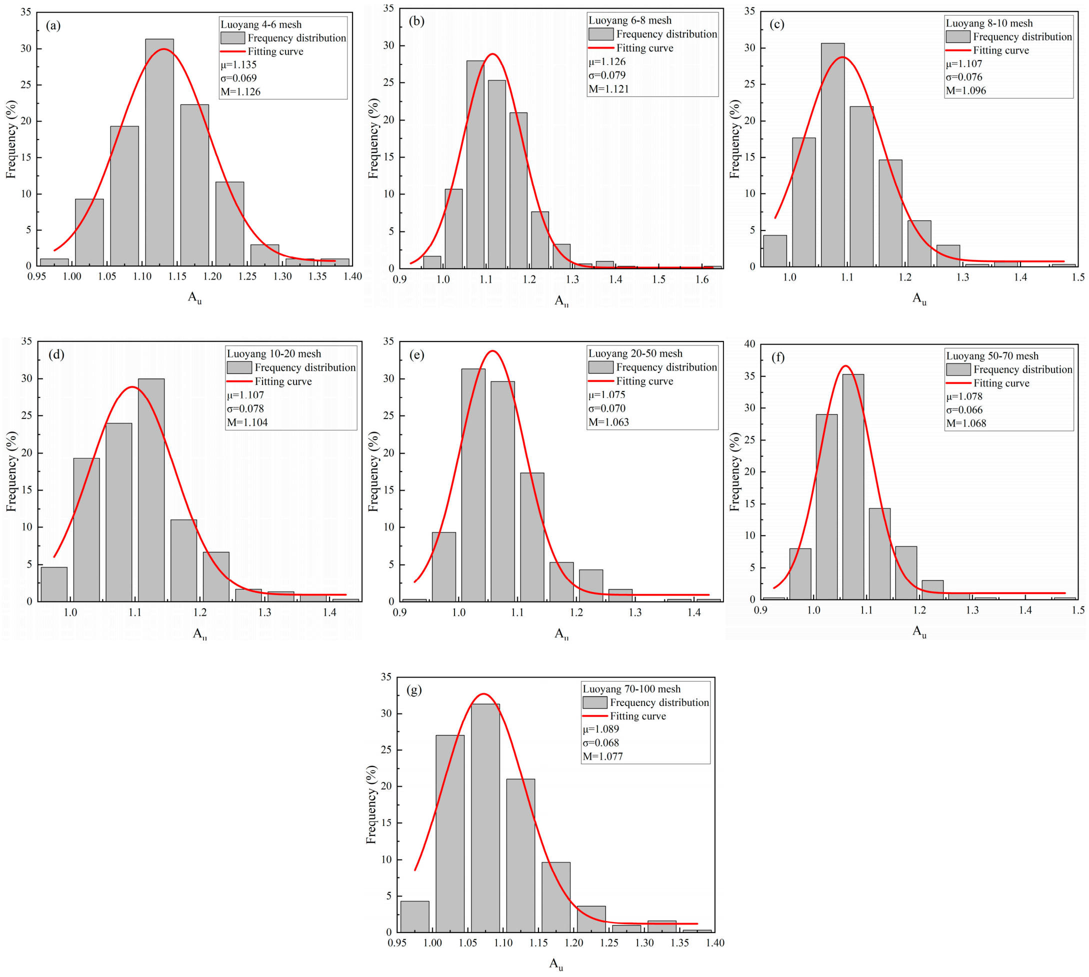

Figure S16. Angularity of quartz particles from Luoyang, Henan.

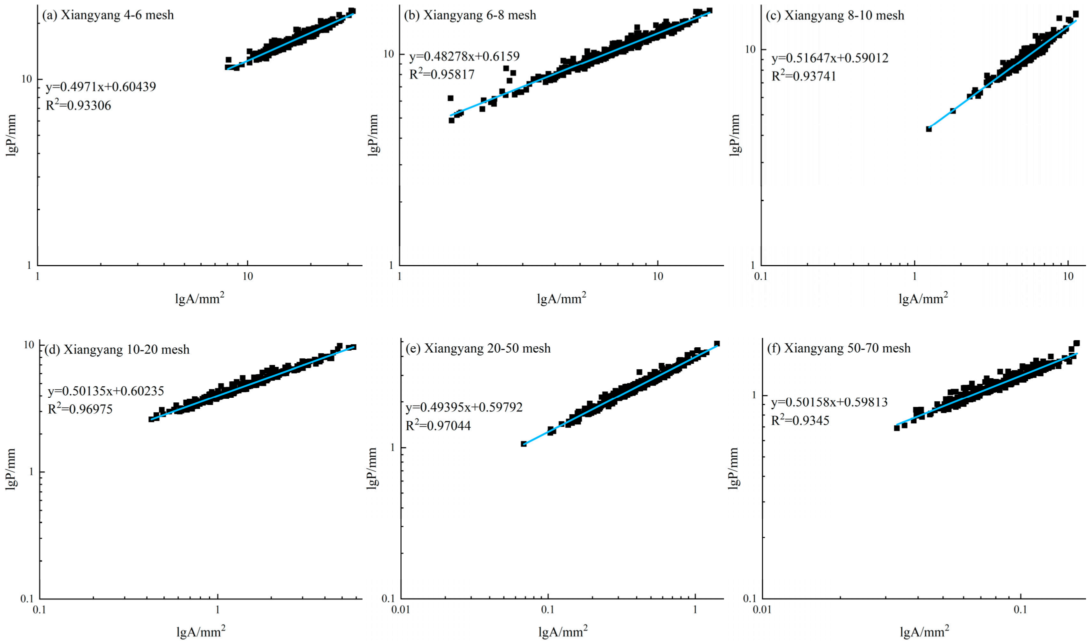

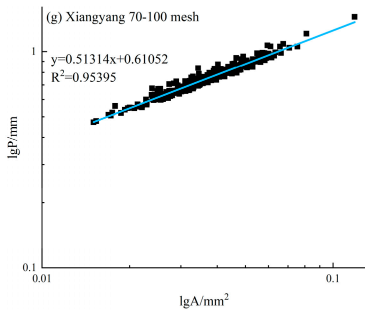

Figure S17. Fitting curves of area (A) and perimeter (P) for quartz particles from Xiangyang, Hubei.

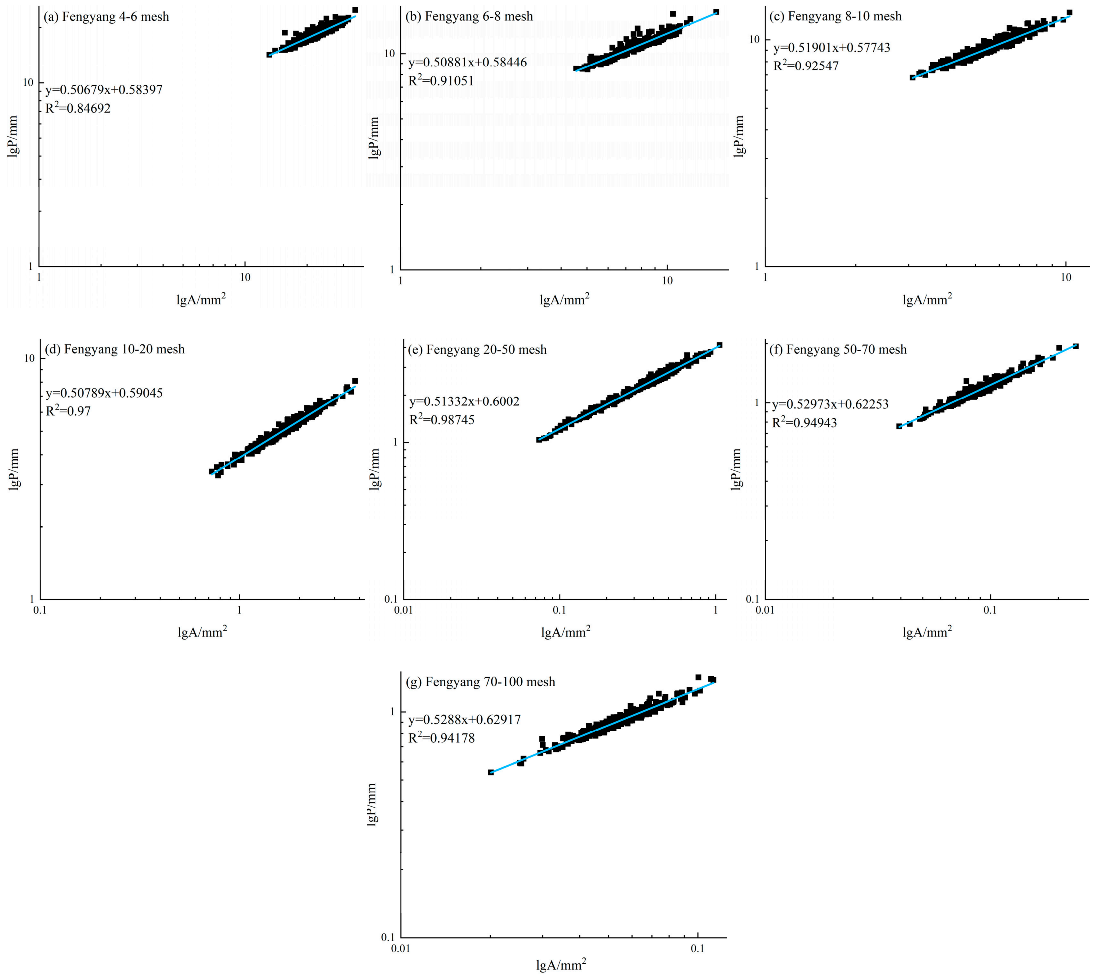

Figure S18. Fitting curves of area (A) and perimeter (P) for quartz particles from Fengyang, Anhui.

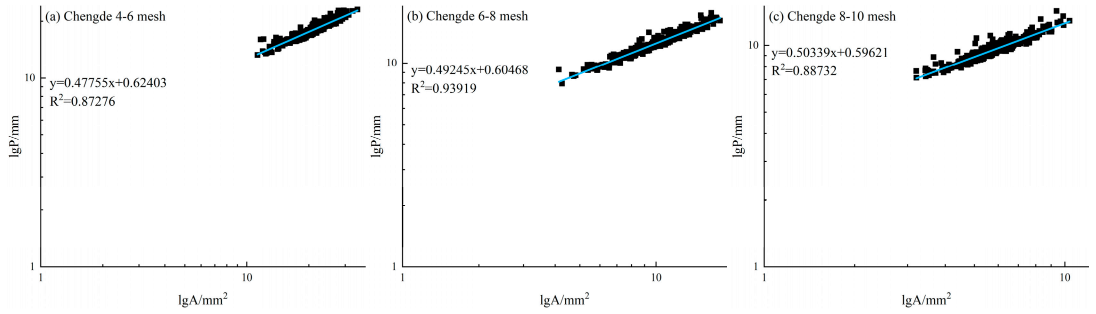

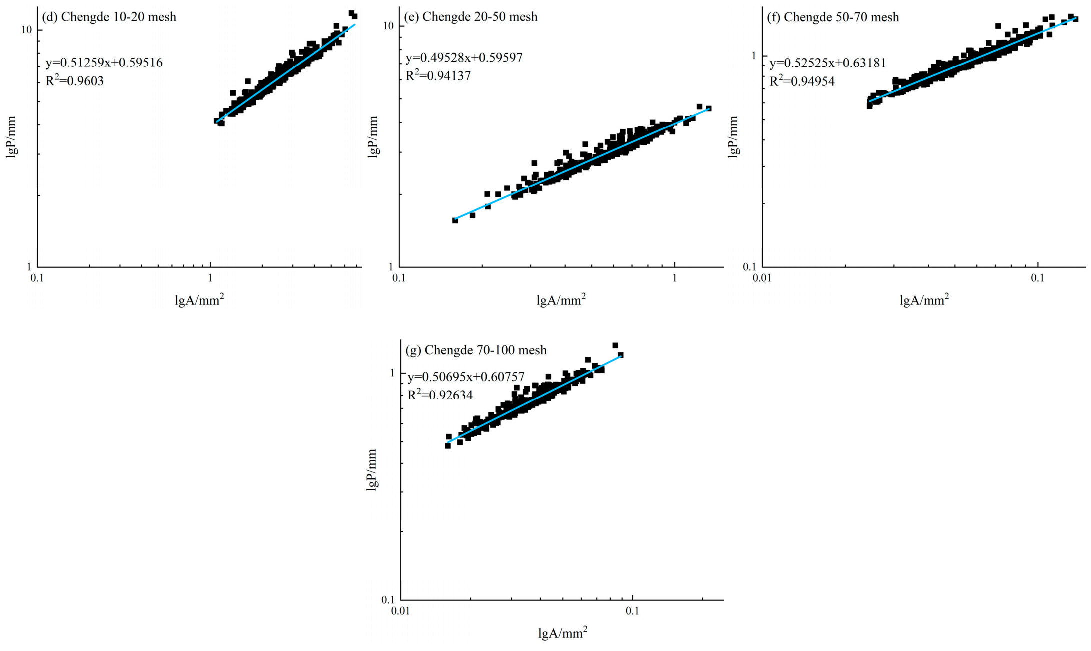

Figure S19. Fitting curves of area (A) and perimeter (P) for quartz particles from Chengde, Hebei.

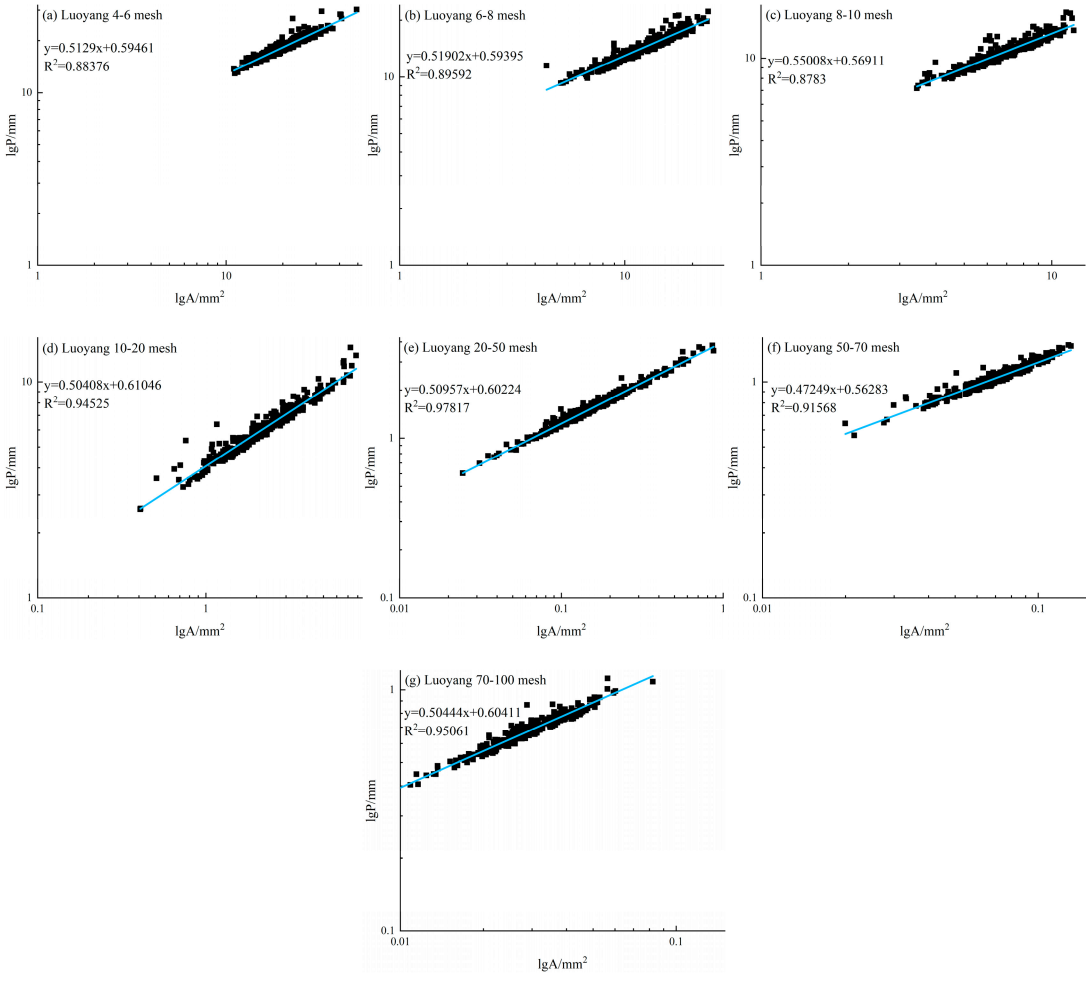

Figure S20. Fitting curves of area (A) and perimeter (P) for quartz particles from Luoyang, Henan.
